# Adherence to initial exclusive breastfeeding among Chinese born and native Spanish mothers

**DOI:** 10.1186/s12884-018-2161-y

**Published:** 2019-01-28

**Authors:** Juana María Aguilar-Ortega, Juan Luis González-Pascual, César Cardenete-Reyes, Carmen Pérez-de-Algaba-Cuenca, Santiago Pérez-García, Laura Esteban-Gonzalo

**Affiliations:** 1 “12 de Octubre” University Hospital, Madrid, Spain; 2 Nursing Department, Rey Juan Carlos University, Alcorcón, Spain; 3 Nursing Department, School of Biomedical and Health Sciences, Universidad Europea de Madrid (UEM), Edificio A. Departamento de Enfermería. c/ Tajo, s/n, 28670 Villaviciosa de Odón, Madrid Spain; 4 Nursing Department, Complutense de Madrid University, Madrid, Spain

**Keywords:** Breastfeeding, Chinese, Immigration, Spain, Postpartum

## Abstract

**Background:**

Benefits of breastfeeding on the health of children, mothers and society are well known. However, breastfeeding rates vary according to the population examined. Chinese-born women migrated to high-income countries have shown low breastfeeding rates. Nevertheless, studies comparing breastfeeding rates of Chinese-born immigrants and natives are scarce.

The aims of this study were therefore: 1) to compare the rate of exclusive breastfeeding at hospital discharge after giving birth between Chinese-born women resident in Spain and native Spanish women, 2) to assess the influence of the biological, socioeconomic, work-related and cultural factors on exclusive breastfeeding in women of Chinese origin.

**Methods:**

A cross-sectional descriptive study with between group comparisons. This study included 73 postpartum women (33 Chinese-born and 40 native Spanish women). The association between exclusive breastfeeding and the country of origin was assessed by binary logistic regression.

**Results:**

Native Spanish women showed a greater prevalence of exclusive breastfeeding (80%) compared to Chinese born immigrant women (36.4%) (adjusted for socioeconomic status, parental level of education, age, cesareans and birth weight) (OR = 0.21; 95% CI 0.05–0.91; *p* = 0.037). However, in other models that considered both work and cultural influences, no differences were observed.

**Discussion:**

The classic biological and socioeconomic variables (educational and socioeconomic levels) do not seem to explain the lower rates of exclusive breastfeeding among Chinese immigrant women. This paradigm of inequity appears to be based on both the work conditions as well as cultural characteristics of Chinese born women in Spain, such as their overall attitude towards breastfeeding.

## Background

In recent years, our knowledge has increased regarding the benefits of breastfeeding on the health of children, mothers and society, both in the short and long term [1]. On the other hand, the inequity that exists regarding breastfeeding between different countries has also come to light (developed versus non-developed countries), as well as inequities within each country (specific groups) [1].

In developed countries, certain groups stand out as having lower breastfeeding rates. For example, this is usually the case of minority groups with a migratory past and/or unfavorable socioeconomic condition. In the United States, the group with the lowest breastfeeding rates are African American women [2]. On an international level, we can highlight the case of Chinese women who have immigrated to Western countries. This latter group presents very low rates of breastfeeding, which are lower than those of native women [3–5]. Chinese-born immigrant women have shown low rates of breastfeeding initiation in Spain: 48% compared to 80% of the Spanish-born women [6].

There are several factors (biological, socioeconomic and cultural) which may influence the practice of breastfeeding. Biological factors include birth by cesarean [7], prematurity [8], low birth weight, smoking mothers [7, 9], the age of the mother and previous problems with breastfeeding [10]. Regarding socioeconomic factors, the socioeconomic level is influential [1], as well as the academic level [11], and the fact that women work outside the home [7], as well as precarious work conditions [12, 13]. Lastly, with regards the cultural factors, we consider the following factors: leave after birth [13, 14], the attitude towards breastfeeding [4, 15–17], prenatal intention to breastfeed [18], transnational maternity [12, 13] and the attitude of significant people in the mother’s surroundings [13, 19].

There is a lack of comparative data regarding the onset of exclusive breastfeeding between native and Chinese women who have immigrated to developed countries.

The aims of this study were therefore: 1) to compare the rate of exclusive breastfeeding at hospital discharge after giving birth between Chinese-born women resident in Spain and native Spanish women, 2) to assess the influence of the biological, socioeconomic, work-related and cultural factors on exclusive breastfeeding in women of Chinese origin.

## Methods

### Study design and data collection

Cross-sectional study with between-group comparison. Data collection was carried out from April to November 2016 as part of a research partnership between the “12 de Octubre” Hospital and the “Europea de Madrid” University regarding breastfeeding [20]. Participants were being admitted at the maternity units of the Hospital after birth. This hospital is located close to the largest Chinese community in Spain, and the one this population is commonly referred to. It is a certified baby-friendly hospital (BFHI), so early initiation of breastfeeding is encouraged for all mothers after birth. Medical staff (nurses, midwifes and others) are exclusively Spanish. Chinese mothers are cared for in the same way as Spanish mothers, although a language barrier exists. The data was gathered using a self-reported survey available in Spanish and Chinese languages (except for feeding method, birth weight and cesarean delivery, which were determined consulting clinical history data). The survey was administered within 48 to 72 h after delivery, while the women were admitted in hospital. A qualified translator was used when necessary.

### Sample

A sample size of 34 participants was estimated for each group, considering a statistical power of 80%, a 95% confidence interval and the previous established data for prevalence of exclusive breastfeeding (48% for Chinese women who have migrated to Spain and 80% in the case of native Spanish women) [6].

The inclusion criteria were postpartum Spanish and Chinese born women. Those mothers with intensive care needs for either themselves or their child, or with any mother/child disease contraindicating breastfeeding or who had an out-of-hospital birth were excluded.

The number of participants in this study was 73 (33 Chinese and 40 Spanish postpartum women). Consecutive sampling was carried out for all Chinese women admitted to the maternity units of the “12 de Octubre” Hospital from April to November 2016 by a researcher in collaboration with the nurses working in the maternity unit. A total of 42 Chinese women were contacted. However, nine Chinese women declined to participate in the study, due to lack of interest or being focused on discharging from the hospital. Every time Chinese-born woman was admitted in the unit and recruited, one Spanish-born woman was randomly selected by a researcher in collaboration with the nurses working in the maternity unit. A total of 43 Spanish women were contacted. However, three declined to participate in the study, for the same reasons as the Chinese women.

### Measurements

#### Breastfeeding

According to WHO, there are three possible feeding methods: exclusive breastfeeding, breastfeeding or bottle-feeding [21]. For the current study, two categories were defined: breast milk (WHO’s exclusive breastfeeding) and other milk. According to WHO, exclusive breastfeeding requires that the infant receive breast milk (including milk expressed or from a wet nurse). The infant is allowed to receive oral rehydration solutions, drops, syrups (vitamins, minerals, medicines) but may not receive anything else [21].

#### Country of birth

Determined by asking participants where they were born.

#### Covariates

Age, socioeconomic status (SES), birth weight, cesarean delivery, paid work (work in exchange for a salary), return-to-work intention, work status (self-employee/public administration employee/private employee), estimated time frame for returning to work, maternal attitudes toward infant feeding methods and intention to send the children to be cared for by other family members outside the home.

Socioeconomic status was assessed by two different methods. First, using the Family Affluence Scale (FAS), a 4-item scale used to measure material wealth, enabling us to stratify women into those with low, medium or high SES [22]. The FAS has been found to reflect family expenditure and consumption, as well as to be reliable and sensitive in differentiating levels of affluence as evidenced by its validation against other measures of SES such as parental occupation and macro-economic indicators at a country level [22]. And second, by recording the self-reported educational level of parents (primary school, secondary school, high school, university).

Maternal attitudes toward infant feeding methods were determined using the Spanish [23] or Chinese [24] version of the Iowa Infant Feeding Attitude Scale (IIFAS). This is a self-reported 17-item questionnaire with a 5-point Likert scale (ranging from strongly disagree to strongly agree). Nine of the items are worded in a manner favorable to breastfeeding, and the remaining eight items are favorable to formula-feeding. Items favoring formula-feeding are reverse-scored. The final score ranges from 17 to 85, where a higher score indicates higher inclination towards breastfeeding. Scores of 70 or higher indicate a positive attitude towards breastfeeding, from 49 to 69 neutral attitude and 48 or lower indicate a positive attitude towards formula feeding. Both the Chinese and Spanish versions of the questionnaire showed an acceptable reliability (Cronbach’s alpha 0.623 and 0.704) and semantic validity (Content Validity Index = 3 or higher). We have contemplated attitudes (influenced by knowledge and believes) as a cultural issue, given that we have considered as a reference a classic broad notion of culture: “that complex whole which includes knowledge, belief, art, law, morals, custom, and any other capabilities and habits acquired by man as a member of society” [25].

### Data analysis

Descriptive statistics are presented using the mean and SD (quantitative variables) or percentage (qualitative variables). Statistical differences between groups (two-sided, *p* < 0.05) were analyzed using Student’s t-test (quantitative variable and qualitative variable) and Chi-squared test (two qualitative variables).

The association between exclusive breastfeeding and the country of origin was assessed by binary logistic regression (odds ratio and 95% confidence interval were calculated). Analyses of interactions were carried out with no significant results. IBM-SPSS Statistics 23.0 was used.

Four different regression models were built with the Spanish mothers as the reference group. Model 1 was adjusted for those biological and socioeconomic variables (SES, age, cesarean and birth weight) with a proven effect on breastfeeding adherence [1, 7, 10], in order to control their impact on our results. Models 2 and 3 were adjusted additionally for work conditions, in order to know their impact on the relation between country of origin and breastfeeding adherence. Thus, model 2 was adjusted for paid work and return to work intention and model 3 was adjusted for more specific work conditions (work status, return to work time frame). Finally, model 4 was adjusted for attitude towards breastfeeding (IIFAS score), with the objective of knowing if this variable could determine the breastfeeding adherence of the Chinese and Spanish women. The goodness of fit was assessed using the Homer-Lemeshow test.

## Results

Native Spanish women showed a greater prevalence of exclusive breastfeeding (80%) compared to Chinese born immigrant women (36.4%) (*p* < 0.001). There was no association between exclusive breastfeeding and length of residence in Spain.

The characteristics of the Chinese and Spanish mothers are shown in Table 1.

**Table 1:** Characteristics of the native Spanish and Chinese immigrant women

	Spanish women	Chinese women	*p* ^a^
N	40	33	
Age [mean, (SD)]	33.1 (5.1)	28.7 (4.3)	**<0.001**
Length of residence in Spain - years [mean, (SD)]		8.7 (4.1)	
Birth weight [mean, (SD)]	3.2 (0.3)	3.5 (0.5)	**0.006**
Cesareans (%)	10.3	13.3	0.692
Mother’s education level (%)			**<0.001**
Primary school	2.5	12.5	
Secondary school	27.5	43.8	
High school	25.0	43.8	
University	45.0	0.0	
Father’s education level (%)			**0.016**
Primary school	7.9	16.1	
Secondary school	18.4	48.4	
High school	36.8	19.4	
University	36.8	16.1	
Socioeconomic status (based on FAS^b^ scores) (%)			0.115
High	27.5	18.8	
Medium	72.5	71.9	
Low	0.0	9.4	
Paid work (%)	73.7	96.7	**0.017**
Return-to-work intention (%)	96.8	100	1.000
Return-to-work time frame –months [mean, (SD)]	5.9 (2.7)	3.9 (1.8)	**0.005**
Work status (%)			**<0.001**
Self-employee	10.7	62.1	
Public administration employee	25.0	3.4	
Employee	64.3	34.5	

^a^Pearsons χ^2^ test (Student’s t-test for age, birth weight and return-to-work time frame). Statistical significance was fitted at two-sided (*p*-value <0.05)

^b^Family Affluence Scale

Bolded *p*-values indicate statistical differences between the means or percentages

Chinese women were younger (*p* < 0.001), worked more outside the home (paid work) (*p* = 0.017), worked more as self-employees (*p* < 0.001), presented a shorter time until resuming work (*p* = 0.005) and showed a lower level of education of both parents (*p* < 0.001 and 0.016, respectively). In the case of the Spanish women, a lower birth weight was registered (*p* = 0.006).

The IIFAS score was lower in Chinese women than in Spanish women (mean 59.3 SD 7.3 versus 71.5 SD 6.9), indicating less breastfeeding-oriented attitudes (*p* < 0.001).

Chinese women showed a greater intention (12.1%) to send the children to be cared for by other family members outside the home than Spanish women (0%) (*p* = 0.025).

Logistic regression models for initial exclusive breastfeeding are presented in Table 2.

**Table 2:** Logistic regression models for exclusive breastfeeding among native Spanish and Chinese immigrant women (*n* = 73)

	Model 1^a^			Model 2^b^			Model 3^c^			Model 4^d^		
	OR	95% CI	p	OR	95% CI	p	OR	95% CI	p	OR	95% CI	p
Spanish women (*n* = 40)	1.00			1.00			1.00			1.00		
Chinese women (*n* = 33)	0.21	0.05–0.90	0.037	0.52	0.06–4.79	0.292	1.68	0.06–44.6	0.852	0.33	0.05–2.33	0.305

^a^Model 1, adjusted for SES (FAS+ parental education level) and biological confounders (age, cesareans, birth weight)

^b^Model 2, adjusted for SES, biological confounders and working conditions (paid work, return-to-work intention)

^c^Model 3, adjusted for SES, biological confounders and working conditions (work status, return-to-work time frame)

^d^Model 4, adjusted for SES, biological confounders and attitudinal variables (IIFAS)

Chinese women had a significantly lower likelihood of initiating exclusive breastfeeding after birth than Spanish women in model 1 (adjusted for SES, parental level of education, age, cesareans and birth weight) (OR = 0.21; 95% CI 0.05–0.91; *p* = 0.037). However, in models 2 (adjusted for SES, parental level of education, age, cesareans, birth weight, paid work and return-to-work intention), 3 (adjusted for SES, parental level of education, age, cesareans, birth weight, estimated time frame for returning to work and work status) and 4 (adjusted for SES, parental level of education, age, cesareans, birth weight, and IIFAS score) there were no differences regarding likelihood of initiating breastfeeding after birth (OR = 0.52; 95% CI 0.06–4.79; *p* = 0.292, for model 2, OR = 1.68; 95% CI 0.06–44.6; *p* = 0.852, for model 3 and OR = 0.33; 95% CI 0.05–2.33; *p* = 0.305, for model 4). The Hosmer–Lemeshow test showed a suitable goodness of fit in all the models, (*p* = 0.610 for model 1, *p* = 0.428 for model 2, *p* = 0.467 for model 3 and *p* = 0.464 for model 4).

## Discussion

### Exclusive breastfeeding in Chinese born mothers

The rate of exclusive breastfeeding at the time of hospital discharge among women of Chinese origin who had immigrated to Spain was significantly lower than that of native women (36.4% versus 80%). This coincides with the results of a previous study in Spain (48% versus 80%) [6] and indicates that this difference has not been corrected with time but rather continues today. This even occurs at a hospital that supports breastfeeding and is certified as a Baby-friendly Hospital. There was no association between exclusive breastfeeding and the length of residence in Spain (as an indirect measure of acculturation).

In Canada, Chinese-origin women showed very low rates (8 to 20%) of breastfeeding which markedly differ from those of the native population - although these have improved over time [3, 5]. In Australia, the oldest data, although much better than reports from Canada and Spain, also reflect a lower rate when compared to the native population (79% versus 91%) [4]. However, the most recent data show very high rates (94.1%) [16], although these cannot be directly compared with those of the native population.

### Breastfeeding and biological and socioeconomic factors

The women of Chinese origin in Spain are five times more likely not to practice exclusive breastfeeding at hospital discharge after giving birth when compared to native women, even after adjusting for biological factors (age of the mother, birth by caesarean, weight at birth) and the socioeconomic and educational levels.

In a previously described study performed in Australia [16], which showed a very high rate of breastfeeding (94.1%), the Chinese women presented a high socioeconomic and educational level (three quarters of the same had studied at university level). In our study, the profile of the immigrant Chinese woman is completely different, with a lower socioeconomic and educational level (none had attended university). Nonetheless, according to a recent systematic review and meta-analysis on women in China, those with a lower educational level have a greater probability of breastfeeding [26].

### Breastfeeding and work-related factors

Regarding the socioeconomic factors related to breastfeeding, it is important to highlight the influence of work factors as being determinant of the adherence to initial exclusive breastfeeding among Chinese women who reside in Spain. This consideration is evident after adjusting for those variables associated with the work status of the women studied (paid work outside the home, the intention of going back to work, the type of work and the estimated time for going back to work after giving birth), which, as a result, meant that the differences previously observed regarding the country of origin stopped being significant, indicating that an over adjustment in the model may have occurred.

In this sense, we have observed that it is more common for women of Chinese origin to work outside the house when compared to native women, however the type of work and the time estimated until they return to work provide a model with an improved explanatory capacity and provide additional details. The women of Chinese origin are mainly self-employed, in contrast with native women who work for the public administration and are employed. Also, the time estimated for going back to work after giving birth is notably lower with a return to work almost 2 months earlier. In Spain, self-employed work is associated with a greater work dedication and a decreased institutional support compared to paid maternity leave. This differs from the conditions observed in Australia, where the proportion of women working full-time was less than 20%. The influence of work on breastfeeding has already been described among the population of Chinese origin [7, 12, 13], however, this is the first study to specify the influence of the job type and the time estimated for returning to work after hospital discharge.

In summary, the observed difference between exclusive breastfeeding at discharge between women of Chinese origin and native women in Spain seems to be partially explained by the work conditions and the difficulty of reconciling family life and work.

### Breastfeeding and cultural factors

When adjusting for cultural factors, such as the attitude towards breastfeeding, in our study, the country of origin is still not significant. The attitude of women of Chinese origin towards breastfeeding is notably less favorable than that of native women. Compared with other studies, the score of the Chinese women in the IIFAS is similar to that of Chinese women in Australia [16] and in China [16, 24]. On the other hand, the score of the native women in the IIFAS is higher when compared to another study performed in Spain [27] and is even higher than that of other Western countries [28–30].

In relation to other cultural aspects, prior qualitative studies have suggested that the intention to send the children to be cared for by other family members outside the home (related with transnational maternity or satellite children) is relevant when attempting to evaluate breastfeeding among women of Chinese origin [12, 13]. However, in the present study, this aspect, although significant in the bivariate analysis, has not shown to be relevant as it is considered an adjustment variable.

### Strengths and limitations

This study presents several limitations. 1) The sample size is modest for carrying out a multivariate analysis. 2) Some variables were collected via mother’s self-reports, which could compromise the quality of the data. 3) All participants were recruited from the same hospital, which may compromise the generalization of results. 4) The cross-sectional study design does not enable the possibility of establishing cause-effect relations.

The present study however, provides new data to the scarce prior literature, especially regarding the factors which could determine the prevalence of exclusive breastfeeding among Chinese immigrants.

## Conclusions

Chinese born women had a lower likelihood of initiating exclusive breastfeeding after birth when compared to native Spanish women in Spain.

The classic biological and socioeconomic variables (educational and socioeconomic levels) do not seem to explain the lower rates of exclusive breastfeeding among Chinese immigrant women. This paradigm of inequity appears to be based on both the work conditions as well as cultural characteristics (attitude towards breastfeeding) of Chinese born women in Spain.

Public health institutions should pay attention to Chinese-born women in Spain. Nurses, midwifes and physicians should probably increase the information provided and improve motivation of the Chinese-born women in order to modify attitudes towards breastfeeding. Implement strategies formulated by consensus with Chinese-born women to facilitate breastfeeding adherence. And also, consider work conditions of these women in order to help them find solutions to initiate or maintain breastfeeding.
